# (*E*)-4-Hy­droxy-*N*′-(3,4,5-trimeth­oxy­benzyl­idene)benzohydrazide

**DOI:** 10.1107/S1600536811041535

**Published:** 2011-10-22

**Authors:** Jirapa Horkaew, Suchada Chantrapromma, Hoong-Kun Fun

**Affiliations:** a Department of Chemistry and Center of Excellence for Innovation in Chemistry, Faculty of Science, Prince of Songkla University, Hat-Yai, Songkhla 90112, Thailand; b Crystal Materials Research Unit, Department of Chemistry, Faculty of Science, Prince of Songkla University, Hat-Yai, Songkhla 90112, Thailand; c X-ray Crystallography Unit, School of Physics, Universiti Sains Malaysia, 11800 USM, Penang, Malaysia

## Abstract

The title benzohydrazide derivative, C_17_H_18_N_2_O_5_, exists in a *trans* conformation with respect to the C=N double bond. The dihedral angle between the benzene rings is 19.41 (5)°. The two meth­oxy groups at the *meta* positions of the trimeth­oxy­benzene group are almost coplanar with the ring [C—O—C—C = 1.62 (16) and 178.33 (10)°], whereas the third meth­oxy group, at the *para* position, is (+)-synclinal with the ring. In the crystal, mol­ecules are linked by N—H⋯O and bifurcated O—H⋯(N,O) hydrogen bonds, as well as weak C—H⋯O inter­actions, into sheets lying parallel to the *ac* plane. A C—H⋯π inter­action also occurs.

## Related literature

For a related structure and background references to benzohydrazide derivatives, see: Fun *et al.* (2011 ▶). For related structures, see: Li & Ban (2009 ▶); Zhang (2011 ▶). For reference bond-length data, see: Allen *et al.* (1987 ▶).
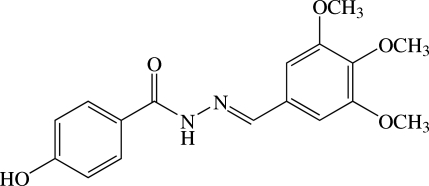


## Experimental

### 

#### Crystal data

C_17_H_18_N_2_O_5_
*M* *_r_* = 330.33
Orthorhombic, 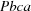

*a* = 14.4623 (8) Å
*b* = 10.9202 (6) Å
*c* = 19.5592 (10) Å
*V* = 3089.0 (3) Å^3^
*Z* = 8
Mo *K*α radiation
μ = 0.11 mm^−1^
*T* = 297 K
0.39 × 0.21 × 0.20 mm

#### Data collection

Bruker SMART APEXII CCD diffractometer
Absorption correction: multi-scan (*SADABS*; Bruker, 2009 ▶) *T*_min_ = 0.960, *T*_max_ = 0.979
20210 measured reflections
4500 independent reflections
3777 reflections with *I* > 2σ(*I*)
*R*_int_ = 0.028

#### Refinement

*R*[*F* ^2^ > 2σ(*F* ^2^)] = 0.039
*wR*(*F* ^2^) = 0.109
*S* = 1.03
4500 reflections
228 parameters
H atoms treated by a mixture of independent and constrained refinement
Δρ_max_ = 0.37 e Å^−3^
Δρ_min_ = −0.32 e Å^−3^

### 

Data collection: *APEX2* (Bruker, 2009 ▶); cell refinement: *SAINT* (Bruker, 2009 ▶); data reduction: *SAINT*; program(s) used to solve structure: *SHELXTL* (Sheldrick, 2008 ▶); program(s) used to refine structure: *SHELXTL*; molecular graphics: *SHELXTL*; software used to prepare material for publication: *SHELXTL* and *PLATON* (Spek, 2009 ▶).

## Supplementary Material

Crystal structure: contains datablock(s) global, I. DOI: 10.1107/S1600536811041535/hb6444sup1.cif

Structure factors: contains datablock(s) I. DOI: 10.1107/S1600536811041535/hb6444Isup2.hkl

Supplementary material file. DOI: 10.1107/S1600536811041535/hb6444Isup3.cml

Additional supplementary materials:  crystallographic information; 3D view; checkCIF report

## Figures and Tables

**Table 1:** Hydrogen-bond geometry (Å, °) *Cg*1 is the centroid of the C1–C6 ring.

*D*—H⋯*A*	*D*—H	H⋯*A*	*D*⋯*A*	*D*—H⋯*A*
O2—H1*O*2⋯O1^i^	0.87 (2)	1.87 (2)	2.6646 (11)	152 (2)
O2—H1*O*2⋯N2^i^	0.87 (2)	2.56 (2)	3.2381 (13)	136.2 (18)
N1—H1*N*1⋯O4^ii^	0.874 (17)	2.088 (17)	2.8891 (12)	152.0 (16)
C6—H6*A*⋯O4^ii^	0.93	2.51	3.4116 (14)	165
C16—H16*B*⋯*Cg*1^iii^	0.96	2.63	3.4572 (17)	145

Symmetry codes: (i) 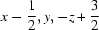; (ii) 
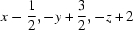
; (iii) 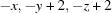.

**Fig. 1.**
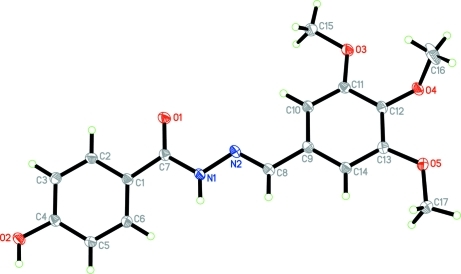
The molecular structure of the title compound, showing 50% probability displacement ellipsoids.

**Fig. 2.**
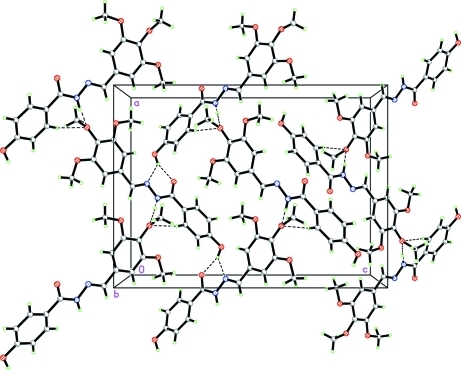
The crystal packing of the title compound viewed along the b axis, Hydrogen bonds were shown as dashed lines.

## supplementary crystallographic information

### Comment

As part of our ongoing studies of benzohydrazide derivatives with possible antibacterial activities (Fun *et al.*, 2011), we now report the synthesis and structure of the title compound, (I). The antibacterial activity of (I) will be reported elsewhere with other related benzohydrazide derivatives.

The molecule of the title benzohydrazide derivative (Fig. 1), C_17_H_18_N_2_O_5_, exists in a *trans*-configuration with respect to the C8═N2 bond [1.2821 (15) Å] and the torsion angle N1–N2–C8–C9 = 178.33 (10)°. The molecule is twisted with the dihedral angle between the two benzene rings being 19.41 (5)°. The middle fragment is slightly twisted which can be indicated by the torsion angle O1–C7–N1–N2 = -6.63 (15)°. The mean plane through this middle bridge (O1/C7/N1/N2/C8) makes the dihedral angles of 12.06 (6) and 8.39 (6)° with the planes of 4-hydroxyphenyl and 3,4,5-trimethoxyphenyl rings, respectively. The three methoxy groups of the 3,4,5-trimethoxyphenyl unit have two different orientations: the two *meta* methoxy groups (at atoms C11 and C13 positions) are co-planar with their attached benzene ring with torsion angles C15–O3–C11–C10 = 1.62 (16)° and C17–O5–C13–C12 = 178.33 (10)° whereas the *para* methoxy is (+)-syn-clinally attached at atom C12 with the torsion angle C16–O4–C12–C11 = 71.28 (15)°. Bond distances are of normal values (Allen *et al.*, 1987) and are comparable with the related structures (Fun *et al.*, 2011; Li & Ban, 2009; Zhang, 2011).

In the crystal packing (Fig. 2), the molecules are linked by N—H···O, O—H···N and O—H···O hydrogen bonds as well as with weak C—H···O interactions (Table 1) into sheets parallel to the *ac* plane. The crystal structure is stabilized by N—H···O, O—H···N, O—H···O hydrogen bonds, weak C—H···O and C—H···π interactions (Table 1).

### Experimental

The title compound (I) was prepared by dissolving 4-hydroxybenzohydrazide (0.1 mmol, 0.15 g) in ethanol (15 ml) and a solution of 3,4,5-trimethoxybenzaldehyde (0.1 mmol, 0.19 g) in ethanol (15 ml) was then added to it0. The mixture was refluxed for around 3 hr and the white solid of the product that appeared was collected by filtration, washed with ethanol and dried in air. Colorless blocks of (I) were obtained after recrystalization from methanol by slow evaporation of the solvent at room temperature after several days, Mp. 532-533 K.

### Refinement

Amide and hydroxy H atoms were located from the difference maps and refined isotropically. The remaining H atoms were positioned geometrically and allowed to ride on their parent atoms, with d(C-H) = 0.93 Å for aromatic and CH and 0.96 Å for CH_3_ atoms. The *U*_iso_ values were constrained to be 1.5*U*_eq_ of the carrier atom for methyl H atoms and 1.2*U*_eq_ for the remaining H atoms. A rotating group model was used for the methyl groups.

### Crystal data

**Table:**

C_17_H_18_N_2_O_5_	*D*_x_ = 1.421 Mg m^−^^3^
*M**_r_* = 330.33	Melting point = 533–532 K
Orthorhombic, *P**b**c**a*	Mo *K*α radiation, λ = 0.71073 Å
Hall symbol: -P 2ac 2ab	Cell parameters from 4500 reflections
*a* = 14.4623 (8) Å	θ = 2.1–30.0°
*b* = 10.9202 (6) Å	µ = 0.11 mm^−^^1^
*c* = 19.5592 (10) Å	*T* = 297 K
*V* = 3089.0 (3) Å^3^	Block, colorless
*Z* = 8	0.39 × 0.21 × 0.20 mm
*F*(000) = 1392	

### Data collection

**Table:**

Bruker SMART APEXII CCD diffractometer	4500 independent reflections
Radiation source: fine-focus sealed tube	3777 reflections with *I* > 2σ(*I*)
graphite	*R*_int_ = 0.028
Detector resolution: 8.33 pixels mm^-1^	θ_max_ = 30.0°, θ_min_ = 2.1°
ω scans	*h* = −18→20
Absorption correction: multi-scan (*SADABS*; Bruker, 2009)	*k* = −15→14
*T*_min_ = 0.960, *T*_max_ = 0.979	*l* = −27→27
20210 measured reflections	

### Refinement

**Table:**

Refinement on *F*^2^	Primary atom site location: structure-invariant direct methods
Least-squares matrix: full	Secondary atom site location: difference Fourier map
*R*[*F*^2^ > 2σ(*F*^2^)] = 0.039	Hydrogen site location: inferred from neighbouring sites
*wR*(*F*^2^) = 0.109	H atoms treated by a mixture of independent and constrained refinement
*S* = 1.03	*w* = 1/[σ^2^(*F*_o_^2^) + (0.0512*P*)^2^ + 1.5827*P*] where *P* = (*F*_o_^2^ + 2*F*_c_^2^)/3
4500 reflections	(Δ/σ)_max_ = 0.001
228 parameters	Δρ_max_ = 0.37 e Å^−^^3^
0 restraints	Δρ_min_ = −0.32 e Å^−^^3^

### Special details

**Table:**

Geometry. All esds (except the esd in the dihedral angle between two l.s. planes) are estimated using the full covariance matrix. The cell esds are taken into account individually in the estimation of esds in distances, angles and torsion angles; correlations between esds in cell parameters are only used when they are defined by crystal symmetry. An approximate (isotropic) treatment of cell esds is used for estimating esds involving l.s. planes.
Refinement. Refinement of F^2^ against ALL reflections. The weighted R-factor wR and goodness of fit S are based on F^2^, conventional R-factors R are based on F, with F set to zero for negative F^2^. The threshold expression of F^2^ > 2sigma(F^2^) is used only for calculating R-factors(gt) etc. and is not relevant to the choice of reflections for refinement. R-factors based on F^2^ are statistically about twice as large as those based on F, and R- factors based on ALL data will be even larger.

### Fractional atomic coordinates and isotropic or equivalent isotropic displacement parameters (Å^2^)

**Table:**

	*x*	*y*	*z*	*U*_iso_*/*U*_eq_	
O1	0.02505 (5)	1.10904 (8)	0.80535 (4)	0.01825 (17)	
O2	−0.32653 (6)	1.14816 (8)	0.61694 (4)	0.01926 (18)	
H1O2	−0.3790 (16)	1.119 (2)	0.6311 (11)	0.051 (6)*	
O3	0.32914 (5)	0.88335 (8)	1.01826 (4)	0.01920 (18)	
O4	0.29967 (5)	0.71610 (8)	1.11552 (4)	0.01747 (17)	
O5	0.13085 (6)	0.63448 (8)	1.14433 (4)	0.01937 (18)	
N1	−0.06135 (6)	0.96981 (9)	0.86248 (5)	0.01526 (18)	
H1N1	−0.1150 (12)	0.9362 (16)	0.8709 (9)	0.029 (4)*	
N2	0.01401 (6)	0.94312 (9)	0.90309 (5)	0.01538 (18)	
C1	−0.12442 (7)	1.07047 (10)	0.76129 (5)	0.0147 (2)	
C2	−0.11500 (7)	1.16838 (11)	0.71558 (6)	0.0165 (2)	
H2A	−0.0627	1.2178	0.7178	0.020*	
C3	−0.18213 (8)	1.19281 (11)	0.66718 (6)	0.0167 (2)	
H3A	−0.1747	1.2578	0.6369	0.020*	
C4	−0.26122 (7)	1.11954 (10)	0.66390 (5)	0.0155 (2)	
C5	−0.26994 (7)	1.01888 (11)	0.70742 (6)	0.0163 (2)	
H5A	−0.3212	0.9678	0.7039	0.020*	
C6	−0.20211 (7)	0.99504 (10)	0.75593 (6)	0.0162 (2)	
H6A	−0.2085	0.9283	0.7851	0.019*	
C7	−0.04843 (7)	1.05222 (10)	0.81117 (5)	0.0142 (2)	
C8	−0.00007 (7)	0.87131 (10)	0.95390 (6)	0.0155 (2)	
H8A	−0.0592	0.8419	0.9630	0.019*	
C9	0.07833 (7)	0.83586 (10)	0.99769 (5)	0.0144 (2)	
C10	0.16646 (7)	0.88419 (10)	0.98592 (5)	0.0154 (2)	
H10A	0.1756	0.9421	0.9517	0.019*	
C11	0.24005 (7)	0.84492 (10)	1.02576 (5)	0.0153 (2)	
C12	0.22580 (7)	0.75942 (10)	1.07816 (5)	0.0152 (2)	
C13	0.13721 (7)	0.71439 (10)	1.09113 (5)	0.0153 (2)	
C14	0.06307 (7)	0.75202 (10)	1.05023 (5)	0.0155 (2)	
H14A	0.0040	0.7213	1.0580	0.019*	
C15	0.34575 (8)	0.97270 (12)	0.96645 (6)	0.0208 (2)	
H15A	0.4092	0.9982	0.9682	0.031*	
H15B	0.3064	1.0422	0.9739	0.031*	
H15C	0.3328	0.9379	0.9224	0.031*	
C16	0.33986 (12)	0.80230 (13)	1.16153 (8)	0.0366 (4)	
H16A	0.4044	0.7842	1.1672	0.055*	
H16B	0.3093	0.7973	1.2050	0.055*	
H16C	0.3330	0.8834	1.1433	0.055*	
C17	0.04118 (8)	0.58866 (12)	1.16066 (6)	0.0221 (2)	
H17A	0.0450	0.5378	1.2006	0.033*	
H17B	0.0181	0.5414	1.1230	0.033*	
H17C	0.0001	0.6560	1.1694	0.033*	

### Atomic displacement parameters (Å^2^)

**Table:**

	*U*^11^	*U*^22^	*U*^33^	*U*^12^	*U*^13^	*U*^23^
O1	0.0125 (3)	0.0233 (4)	0.0190 (4)	−0.0017 (3)	0.0000 (3)	0.0030 (3)
O2	0.0142 (4)	0.0264 (4)	0.0172 (4)	−0.0013 (3)	−0.0031 (3)	0.0046 (3)
O3	0.0124 (4)	0.0266 (4)	0.0186 (4)	−0.0017 (3)	−0.0006 (3)	0.0051 (3)
O4	0.0140 (3)	0.0186 (4)	0.0197 (4)	0.0021 (3)	−0.0051 (3)	0.0012 (3)
O5	0.0163 (4)	0.0222 (4)	0.0197 (4)	−0.0015 (3)	−0.0027 (3)	0.0078 (3)
N1	0.0108 (4)	0.0191 (4)	0.0158 (4)	−0.0002 (3)	−0.0020 (3)	0.0032 (3)
N2	0.0124 (4)	0.0185 (4)	0.0152 (4)	0.0024 (3)	−0.0027 (3)	−0.0001 (3)
C1	0.0121 (4)	0.0184 (5)	0.0135 (4)	0.0012 (4)	0.0003 (3)	0.0009 (4)
C2	0.0139 (5)	0.0193 (5)	0.0164 (5)	−0.0026 (4)	0.0002 (4)	0.0015 (4)
C3	0.0164 (5)	0.0187 (5)	0.0150 (5)	−0.0016 (4)	0.0001 (4)	0.0033 (4)
C4	0.0134 (4)	0.0197 (5)	0.0134 (4)	0.0016 (4)	0.0004 (4)	0.0000 (4)
C5	0.0132 (4)	0.0180 (5)	0.0176 (5)	−0.0014 (4)	−0.0002 (4)	0.0012 (4)
C6	0.0147 (5)	0.0170 (5)	0.0167 (5)	0.0003 (4)	0.0003 (4)	0.0031 (4)
C7	0.0124 (4)	0.0167 (5)	0.0134 (4)	0.0021 (4)	0.0011 (3)	−0.0001 (4)
C8	0.0132 (4)	0.0177 (5)	0.0157 (5)	0.0007 (4)	−0.0011 (4)	−0.0008 (4)
C9	0.0135 (4)	0.0157 (5)	0.0138 (4)	0.0017 (4)	−0.0009 (3)	−0.0015 (4)
C10	0.0142 (5)	0.0182 (5)	0.0139 (4)	0.0002 (4)	0.0003 (4)	0.0008 (4)
C11	0.0124 (4)	0.0184 (5)	0.0152 (4)	0.0002 (4)	0.0008 (4)	−0.0010 (4)
C12	0.0132 (4)	0.0173 (5)	0.0151 (4)	0.0028 (4)	−0.0016 (3)	0.0001 (4)
C13	0.0160 (5)	0.0163 (5)	0.0136 (4)	0.0010 (4)	−0.0010 (4)	0.0005 (4)
C14	0.0132 (4)	0.0172 (5)	0.0160 (5)	0.0000 (4)	−0.0006 (4)	0.0002 (4)
C15	0.0168 (5)	0.0258 (6)	0.0199 (5)	−0.0023 (4)	0.0012 (4)	0.0046 (4)
C16	0.0504 (9)	0.0268 (7)	0.0326 (7)	0.0135 (6)	−0.0256 (7)	−0.0118 (6)
C17	0.0182 (5)	0.0249 (6)	0.0233 (6)	−0.0036 (4)	−0.0010 (4)	0.0079 (5)

### Geometric parameters (Å, °)

**Table:**

O1—C7	1.2358 (13)	C5—H5A	0.9300
O2—C4	1.3542 (13)	C6—H6A	0.9300
O2—H1O2	0.87 (2)	C8—C9	1.4727 (14)
O3—C11	1.3630 (13)	C8—H8A	0.9300
O3—C15	1.4270 (14)	C9—C14	1.3939 (15)
O4—C12	1.3780 (12)	C9—C10	1.3986 (15)
O4—C16	1.4262 (15)	C10—C11	1.3871 (15)
O5—C13	1.3611 (13)	C10—H10A	0.9300
O5—C17	1.4262 (14)	C11—C12	1.4016 (15)
N1—C7	1.3608 (14)	C12—C13	1.3957 (15)
N1—N2	1.3797 (12)	C13—C14	1.3995 (15)
N1—H1N1	0.874 (18)	C14—H14A	0.9300
N2—C8	1.2821 (15)	C15—H15A	0.9600
C1—C6	1.3971 (15)	C15—H15B	0.9600
C1—C2	1.4004 (15)	C15—H15C	0.9600
C1—C7	1.4831 (14)	C16—H16A	0.9600
C2—C3	1.3820 (15)	C16—H16B	0.9600
C2—H2A	0.9300	C16—H16C	0.9600
C3—C4	1.3974 (15)	C17—H17A	0.9600
C3—H3A	0.9300	C17—H17B	0.9600
C4—C5	1.3959 (15)	C17—H17C	0.9600
C5—C6	1.3894 (15)		
			
C4—O2—H1O2	108.0 (14)	C10—C9—C8	120.44 (10)
C11—O3—C15	116.49 (9)	C11—C10—C9	119.34 (10)
C12—O4—C16	115.09 (9)	C11—C10—H10A	120.3
C13—O5—C17	117.23 (9)	C9—C10—H10A	120.3
C7—N1—N2	117.12 (9)	O3—C11—C10	124.72 (10)
C7—N1—H1N1	122.6 (11)	O3—C11—C12	115.01 (9)
N2—N1—H1N1	120.2 (11)	C10—C11—C12	120.26 (10)
C8—N2—N1	116.75 (9)	O4—C12—C13	119.62 (10)
C6—C1—C2	118.72 (10)	O4—C12—C11	120.16 (9)
C6—C1—C7	124.48 (10)	C13—C12—C11	120.17 (10)
C2—C1—C7	116.77 (10)	O5—C13—C12	115.27 (9)
C3—C2—C1	121.09 (10)	O5—C13—C14	125.00 (10)
C3—C2—H2A	119.5	C12—C13—C14	119.73 (10)
C1—C2—H2A	119.5	C9—C14—C13	119.54 (10)
C2—C3—C4	119.73 (10)	C9—C14—H14A	120.2
C2—C3—H3A	120.1	C13—C14—H14A	120.2
C4—C3—H3A	120.1	O3—C15—H15A	109.5
O2—C4—C5	122.16 (10)	O3—C15—H15B	109.5
O2—C4—C3	118.03 (10)	H15A—C15—H15B	109.5
C5—C4—C3	119.79 (10)	O3—C15—H15C	109.5
C6—C5—C4	120.01 (10)	H15A—C15—H15C	109.5
C6—C5—H5A	120.0	H15B—C15—H15C	109.5
C4—C5—H5A	120.0	O4—C16—H16A	109.5
C5—C6—C1	120.57 (10)	O4—C16—H16B	109.5
C5—C6—H6A	119.7	H16A—C16—H16B	109.5
C1—C6—H6A	119.7	O4—C16—H16C	109.5
O1—C7—N1	121.20 (10)	H16A—C16—H16C	109.5
O1—C7—C1	120.61 (10)	H16B—C16—H16C	109.5
N1—C7—C1	118.18 (9)	O5—C17—H17A	109.5
N2—C8—C9	119.29 (10)	O5—C17—H17B	109.5
N2—C8—H8A	120.4	H17A—C17—H17B	109.5
C9—C8—H8A	120.4	O5—C17—H17C	109.5
C14—C9—C10	120.90 (10)	H17A—C17—H17C	109.5
C14—C9—C8	118.65 (10)	H17B—C17—H17C	109.5
			
C7—N1—N2—C8	175.78 (10)	C8—C9—C10—C11	−176.91 (10)
C6—C1—C2—C3	1.83 (17)	C15—O3—C11—C10	1.62 (16)
C7—C1—C2—C3	179.98 (10)	C15—O3—C11—C12	−178.52 (10)
C1—C2—C3—C4	0.52 (17)	C9—C10—C11—O3	178.62 (10)
C2—C3—C4—O2	178.38 (10)	C9—C10—C11—C12	−1.23 (16)
C2—C3—C4—C5	−2.91 (17)	C16—O4—C12—C13	−111.02 (14)
O2—C4—C5—C6	−178.41 (10)	C16—O4—C12—C11	71.28 (15)
C3—C4—C5—C6	2.94 (16)	O3—C11—C12—O4	−2.99 (15)
C4—C5—C6—C1	−0.57 (17)	C10—C11—C12—O4	176.88 (10)
C2—C1—C6—C5	−1.80 (16)	O3—C11—C12—C13	179.33 (10)
C7—C1—C6—C5	−179.80 (10)	C10—C11—C12—C13	−0.80 (17)
N2—N1—C7—O1	−6.63 (15)	C17—O5—C13—C12	178.33 (10)
N2—N1—C7—C1	172.15 (9)	C17—O5—C13—C14	−1.93 (16)
C6—C1—C7—O1	167.40 (11)	O4—C12—C13—O5	4.06 (15)
C2—C1—C7—O1	−10.63 (15)	C11—C12—C13—O5	−178.24 (10)
C6—C1—C7—N1	−11.39 (16)	O4—C12—C13—C14	−175.69 (10)
C2—C1—C7—N1	170.58 (10)	C11—C12—C13—C14	2.00 (16)
N1—N2—C8—C9	177.83 (9)	C10—C9—C14—C13	−0.90 (16)
N2—C8—C9—C14	−176.20 (10)	C8—C9—C14—C13	178.12 (10)
N2—C8—C9—C10	2.83 (16)	O5—C13—C14—C9	179.12 (10)
C14—C9—C10—C11	2.10 (16)	C12—C13—C14—C9	−1.15 (16)

### Hydrogen-bond geometry (Å, °)

**Table:**

*Cg*1 is the centroid of the C1–C6 ring.

**Table:**

*D*—H···*A*	*D*—H	H···*A*	*D*···*A*	*D*—H···*A*
O2—H1O2···O1^i^	0.87 (2)	1.87 (2)	2.6646 (11)	152 (2)
O2—H1O2···N2^i^	0.87 (2)	2.56 (2)	3.2381 (13)	136.2 (18)
N1—H1N1···O4^ii^	0.874 (17)	2.088 (17)	2.8891 (12)	152.0 (16)
C6—H6A···O4^ii^	0.93	2.51	3.4116 (14)	165
C16—H16B···Cg1^iii^	0.96	2.63	3.4572 (17)	145

Symmetry codes: (i) *x*−1/2, *y*, −*z*+3/2; (ii) *x*−1/2, −*y*+3/2, −*z*+2; (iii) −*x*, −*y*+2, −*z*+2.
